# Post-Endoscopy Parotid Swelling: A Rare Complication

**DOI:** 10.7759/cureus.54804

**Published:** 2024-02-24

**Authors:** Kamlesh Taori, Vijendra Kirnake, Vishal Padwale, Rupal Laddha, Parmeshwar R Junare

**Affiliations:** 1 Gastroenterology, Jawaharlal Nehru Medical College, Datta Meghe Institute of Higher Education & Research, Wardha, IND; 2 Medicine, Jawaharlal Nehru Medical College, Datta Meghe Institute of Higher Education & Research, Wardha, IND; 3 Gastroenterology, Topiwala National Medical College and Bai Yamunabai Laxman Nair Charitable Hospital, Mumbai, IND

**Keywords:** etiology, mechanical trauma, endoscopic procedure, parotid swelling, post-endoscopy complication

## Abstract

Parotid gland swelling, or parotitis, typically associated with infectious causes, can uncommonly result from non-infectious factors such as mechanical trauma following endoscopic procedures. We present a case of a 46-year-old female with liver cirrhosis who developed right parotid swelling shortly after undergoing endoscopy for evaluation of gastrointestinal symptoms. The patient’s clinical course, imaging findings, and successful resolution with conservative measures are detailed. The etiology of post-endoscopy parotid swelling is multifactorial, involving potential mechanisms such as mechanical trauma, salivary gland dysfunction, infection, ductal obstruction, or allergic reactions to medications. Diagnosing this rare complication requires a comprehensive clinical evaluation, including a detailed history, symptom assessment, and imaging studies such as ultrasound. Management involves a combination of symptomatic relief, identification, and treatment of the underlying cause, emphasizing the importance of early recognition to prevent complications. In our case, warm compression provided pain relief, and the swelling subsided without the need for medical or surgical intervention. Regular follow-up evaluations and imaging studies are crucial to assess treatment response and ensure the resolution of the swelling. This case contributes to the limited literature on post-endoscopy parotid swelling, emphasizing the significance of recognizing and managing this rare complication promptly. Healthcare professionals should be vigilant, and further research is encouraged to better understand its pathophysiology and optimize management strategies in order to improve patient outcomes.

## Introduction

Parotid gland swelling, also known as parotitis, is characterized by inflammation and enlargement of the parotid gland, the largest salivary gland on either side of the face, just below the ear [1]. While infectious causes, such as viral or bacterial infections, are the most common etiologies of parotid swelling, non-infectious causes, including mechanical trauma and ductal obstruction, should also be considered in the differential diagnosis [2]. Endoscopy is a minimally invasive procedure commonly used in various medical specialties for diagnostic and therapeutic purposes. Although endoscopic procedures are generally considered safe, complications can still occur, and parotid swelling following endoscopy is an infrequent but recognized complication [3]. The reported incidence of this complication varies in the literature, with limited case reports available to guide clinicians in its diagnosis and management. In this case report, we present a case of a female who developed parotid swelling shortly after undergoing an endoscopic procedure. We aim to highlight the importance of recognizing and managing this unusual complication and discuss possible underlying mechanisms. By reporting and analyzing this case, we hope to contribute to the knowledge of post-endoscopy parotid swelling, raise awareness among healthcare professionals, and encourage further research to better understand its pathophysiology and optimize management strategies. Written and oral informed consent was obtained from individuals who participated in the study to document this case report.

## Case presentation

A 46-year-old female with a known case of cirrhosis, non-B, non-C, and a Child-Pugh score of 8 (CTP-B) for the liver with portal hypertension, bicytopenia, ascites, esophageal varices, and portal hypertensive gastropathy (PGP), without hepatic encephalopathy or hepatorenal syndrome, presented to the gastroenterology OPD. She complained of easy fatiguability, loss of appetite, and abdominal distension for one month. Additionally, she had a history of passing black stools on and off for the last 15 days. Table 1 shows the laboratory investigation. The stool test for occult blood was positive.

**Table 1:** Laboratory investigation reports INR, international normalized ratio

Variable	Value	Normal range
Hemoglobin	7.9 gm%	12-15 gm%
Leucocyte count	4,800/mm^3^	4,000-10,000/mm^3^
Platelets	61,000/mm^3^	150,000-410,000/mm^3^
Coagulopathy (INR)	1.79	
Hypoalbuminemia	2.8 g/dL	3.5-5 g/dL
Serum amylase level	78 mg/dL	40-140 mg/dL

**Figure 1:**
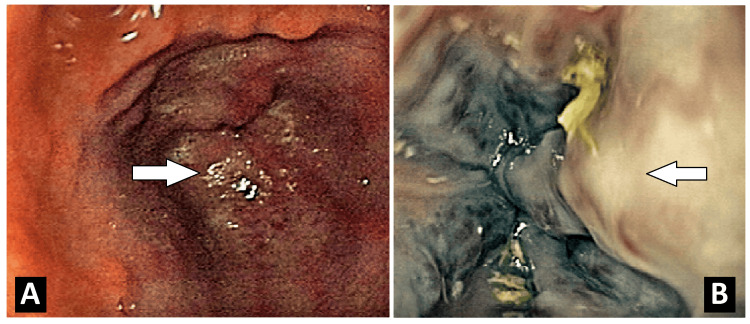
Endoscopic image showing large esophageal varices: (A) GAVE. (B) Mild PGP. GAVE, gastric antral vascular ectasia; PGP, portal hypertensive gastropathy

To find out the cause of anemia and black stools, the patient underwent an endoscopy. The procedure was explained, and consent was obtained. A local anesthetic, lignocaine spray, was given before the procedure. A Flexible HD Olympus CV-170 Video Endoscope (Olympus Corporation, Tokyo, Japan) was used, revealing large esophageal varices with mild PGP and gastric antral vascular ectasia. Endoscopic band ligature for large esophageal varices was performed (Figure 1).

**Figure 2:**
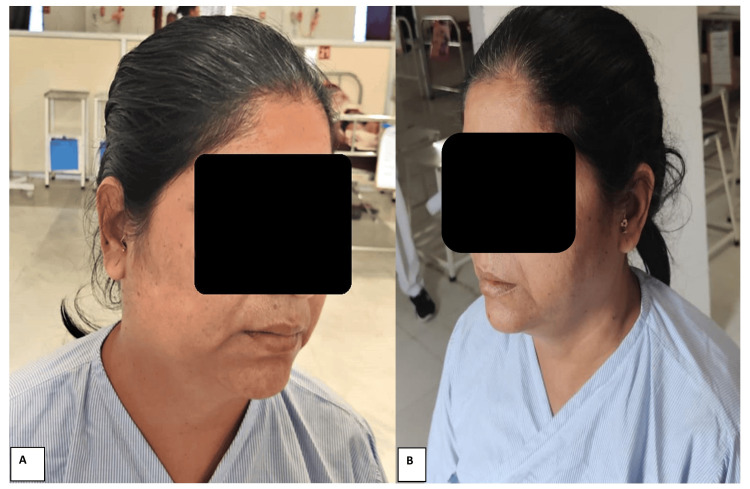
Right parotid swelling

Five minutes after the procedure, the patient developed right parotid swelling and pain. The pain was of sudden onset, dull aching that aggravated on chewing and opening of the mouth, without any radiation. On local examination, she had a very mild erythematous swelling in the parotid region of the right side (Figure 2). It was soft and tender, with no rise in the local temperature. It was not associated with excessive salivation or gingival hyperplasia. However, otherwise, she was clinically normal.

**Figure 3:**
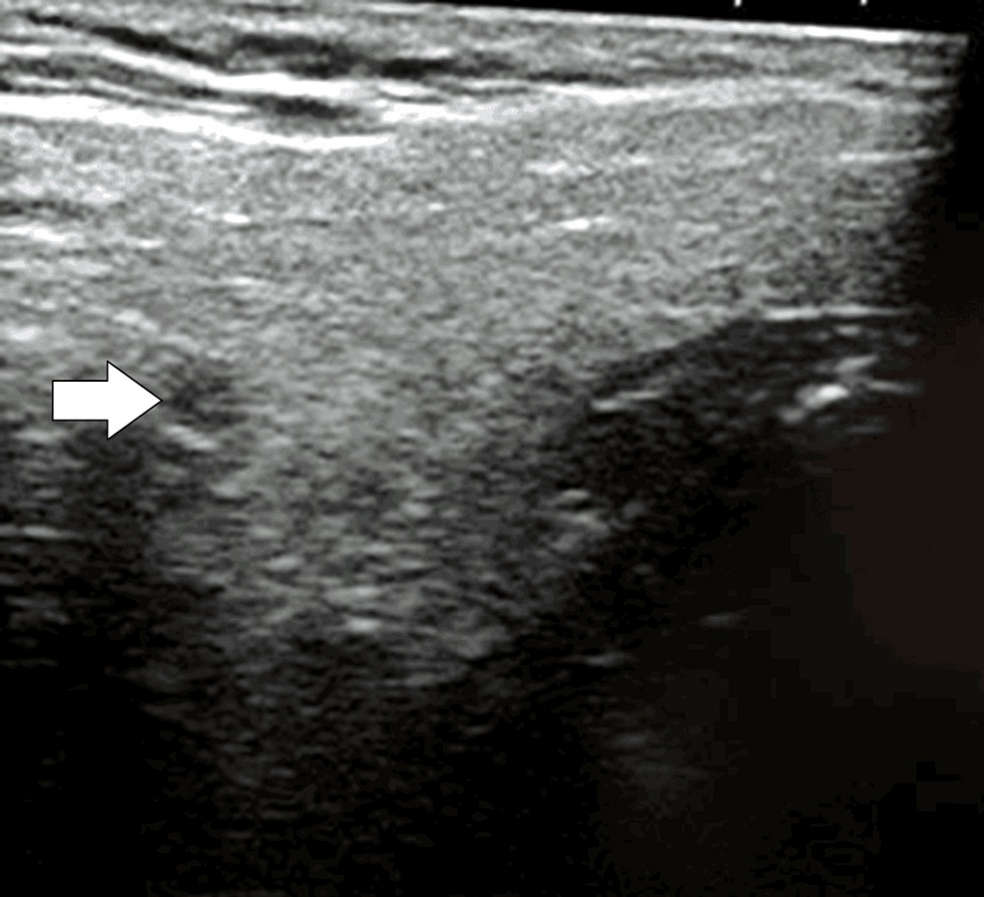
Ultrasonography showing a bulky parotid without any rise in vascularity

Ultrasonography of local parotid swelling revealed a bulky parotid without any rise in vascularity (Figure 3).

For pain relief, the patient was advised to seek relief through warm compression. The swelling subsided after four to six hours. No medical or surgical intervention was required. The patient was closely monitored for another 48 hours. The later course was uneventful, and the patient was discharged.

## Discussion

Post-endoscopy parotid swelling is a rare complication that can occur following various endoscopic procedures [3]. Although the exact pathophysiology and incidence of this complication are not well established, it is important to recognize and manage it appropriately to prevent potential complications and improve patient outcomes. The etiology of post-endoscopy parotid swelling can be multifactorial. It may result from direct mechanical trauma to the parotid gland during the procedure, leading to inflammation and subsequent swelling [4]. Additionally, the insertion of endoscopic instruments into the oral cavity can cause transient salivary gland dysfunction, leading to salivary stasis and subsequent swelling [4]. Other potential mechanisms include infection, ductal obstruction, or an allergic reaction to medications used during the procedure [4]. To diagnose post-endoscopy parotid swelling, a thorough clinical evaluation is necessary. This may include a detailed history of the endoscopic procedure, an assessment of the patient’s symptoms, and a physical examination of the affected area. Imaging studies such as ultrasound, CT, or MRI may help evaluate the parotid gland and rule out other causes of swelling, such as abscess formation or tumors [2].

The management of post-endoscopy parotid swelling involves a combination of symptomatic relief, identification and treatment of the underlying cause, and close monitoring of the patient’s clinical progress. Symptomatic relief can be achieved by applying warm compresses and using analgesics to alleviate pain and inflammation. In cases where an infectious etiology is suspected, appropriate antimicrobial therapy should be initiated. If ductal obstruction is present, gentle massage, warm compresses, and sialogogues may help promote the passage of saliva and relieve the obstruction. Surgical intervention, such as drainage of an abscess or ductal dilatation, may be necessary in certain cases. In this case report, we present a patient who developed significant swelling of the parotid gland following an esophagogastroduodenoscopy procedure. The swelling occurred within minutes after the procedure and was accompanied by pain. Imaging studies confirmed the presence of parotid gland enlargement without evidence of ductal obstruction or other underlying pathology.

It is crucial to emphasize the importance of early recognition and management of post-endoscopy parotid swelling to prevent complications such as abscess formation, ductal strictures, or chronic parotid dysfunction. Regular follow-up evaluations and imaging studies should be considered to assess the response to treatment and ensure the resolution of the swelling. While limited literature specifically addresses post-endoscopy parotid swelling, a few case reports document similar cases. This complication can also occur after a procedure like bronchoscopy [5] or endotracheal intubation [6]. For example, a case report by Bailey described a patient who developed parotid swelling after undergoing a bronchoscopy procedure. The author attributed the swelling to ductal obstruction caused by pressure exerted by the endoscope during the procedure [7]. Another case report by Franco et al. reported a patient who developed parotid swelling following endoscopic sinus surgery, and the authors speculated that trauma to the parotid duct during the procedure might have contributed to the swelling [8].

While limited literature specifically addresses post-endoscopy parotid swelling, a few case reports document similar cases. This complication can also occur after a procedure like bronchoscopy [5] or endotracheal intubation [6]. A case report by Li and Chahal described a patient who developed parotid swelling after undergoing an ERCP procedure. They could not find the exact etiology of post-ERCP swelling; however, the authors speculated that trauma to the parotid duct during the procedure might have contributed to the swelling [9]. Another case report by Chidipothu et al. reported a 41-year-old patient who developed acute bilateral swelling of the parotid gland after general anesthesia in the lateral decubitus position, which subsided after 24-48 hours with intravenous steroid injections [10]. Molakatalla and Govil reported right submandibular swelling five hours after 54-French bougie dilatation, which resolved after 72 hours of conservative management [11]. It is essential for endoscopists to be aware of the possibility of parotid swelling as a complication of endoscopic procedures and to take precautions to minimize the risk. This includes careful insertion and manipulation of the endoscope and consideration of alternative techniques or approaches in patients with known risk factors such as salivary gland disease or prior radiation therapy.

## Conclusions

Post-endoscopy parotid swelling is a rare complication that can occur following various endoscopic procedures. While the exact mechanism remains unclear, it is believed to involve trauma or compression of the parotid gland during the insertion or manipulation of the endoscope. Management is typically supportive, with most cases resolving spontaneously within a few days to weeks. However, surgical or medical interventions may be necessary in rare instances where the swelling persists or is associated with complications. Endoscopists should be aware of this potential complication and take appropriate precautions to minimize the risk, particularly in patients with known risk factors or anatomical variations of the salivary glands.
